# Epithelial-myoepithelial carcinoma of the tongue base: a case for the case-report and review of the literature

**DOI:** 10.1186/1758-3284-2-4

**Published:** 2010-02-02

**Authors:** Peter Peters, Costa Repanos, James Earnshaw, Patrick Stark, Bryan Burmeister, Lloyd McGuire, Susanne Jeavons, William B Coman AM

**Affiliations:** 1 Department of Otolaryngology Head and Neck Surgery, Princess Alexandra Hospital, Ipswich Rd, Woolloongabba, Queensland, Australia; 2 Department of Radiation Oncology, Princess Alexandra Hospital, Ipswich Rd, Woolloongabba, Queensland, Australia; 3 School of Medicine, University of Queensland, St Lucia, Queensland, Australia; 4 Queensland Health Pathology Service, Gold Coast Hospital, Nerang St, Southport, Queensland, Australia; 5 Faculty of Health Sciences and Medicine, Bond University, Robina, Queensland, Australia; 6 Radiology Department, Princess Alexandra Hospital, Ipswich Rd, Woolloongabba, Queensland, Australia

## Abstract

A 60 year old lady was referred to the Princess Alexandra Hospital (Brisbane, Queensland, Australia) tertiary Otolaryngology, Head and Neck Unit from a peripheral hospital for investigation and management of a tumour at the base of the tongue. Biopsy of the tumour revealed it to be an epithelial-myoepithelial carcinoma of the base of the tongue. This is an extremely rare tumour in this location with only 2 other case reports in the world literature: the patients were treated with chemo-radiotherapy and surgery respectively. Our patient was made aware of the world literature and was able to make a fully informed decision on her choice of treatment modality and was treated with radiotherapy. Increasingly journals are limiting publication of case reports to "world firsts" only. We present a case where such a policy would have denied patient choice and possibly led to detrimental treatment.

We review the world literature of tongue base epithelial-myoepithelial carcinoma of the tongue.

## Introduction

Epithelial-myoepithelial carcinoma is an rare tumour occurring in the salivary glands, (most commonly in the parotid gland), with a reported incidence of between 0.2% [1] and 1% [2,3] of salivary duct tumours. It is a hybrid tumour, composed of two different tumour entities, each of which conforms to a defined tumour category [4,5] and based upon the Salivary Gland Register at the University of Hamburg. Specimens collected between 1965 and 1994 showed that only 0.1% of salivary gland tumours were hybrid tumours [5]. Other examples of hybrid tumours include mucoepidermoid carcinoma, basaloid-squamous carcinoma, adeno-squamous carcinoma, sarcomatoid carcinoma and carcinoma in pleomorphic adenoma with differentiation as squamous cell carcinoma as well as adenocarcinoma [5].

## Case Report

A 60 year old lady was referred to the Princess Alexandra Hospital Head and Neck Clinic in July 2009 following a biopsy taken from a suspected tumour at the base of the tongue (BOT). The patient was referred to our unit for ongoing investigation and management. She had noted a one year history of dysphagia and difficulty in moving her tongue. Our patient also noted an unintended a 25 kg weight loss over the past year and had had a PEG feeding tube inserted in the interim before being referred to our department. The patient confirmed that she was an ex-smoker with a 30 pack year history but denied alcohol consumption.

**Figure 1:**
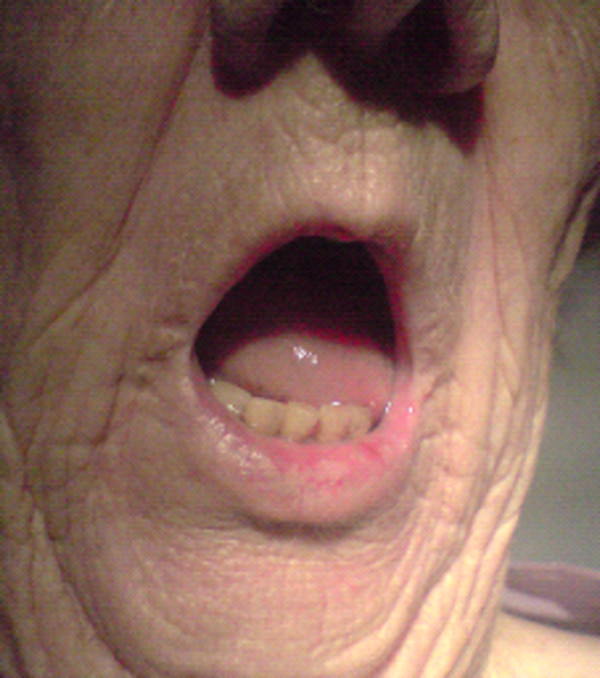
**Extent of limited tongue protrusion due to tumour invasion of tongue muscles**.

**Figure 2:**
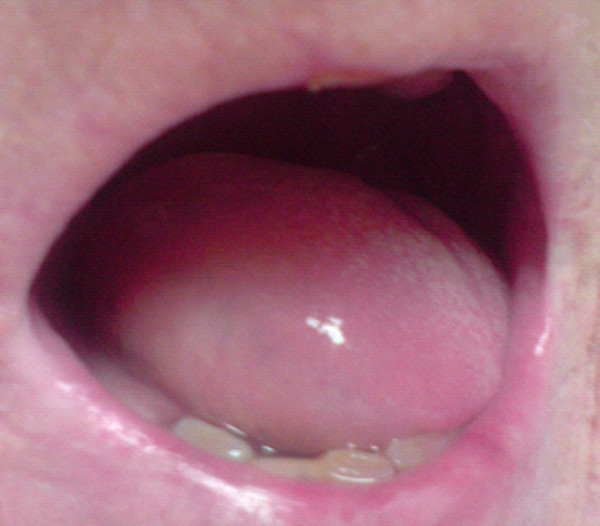
**Close up of the tongue, with the patient protruding tongue maximally**.

On examination there was limited movement of her tongue with significant tethering (Figure 1 and 2). A large mass was palpable predominantly on the right side of the base of the tongue and there were no masses palpable in the neck.

**Figure 3:**
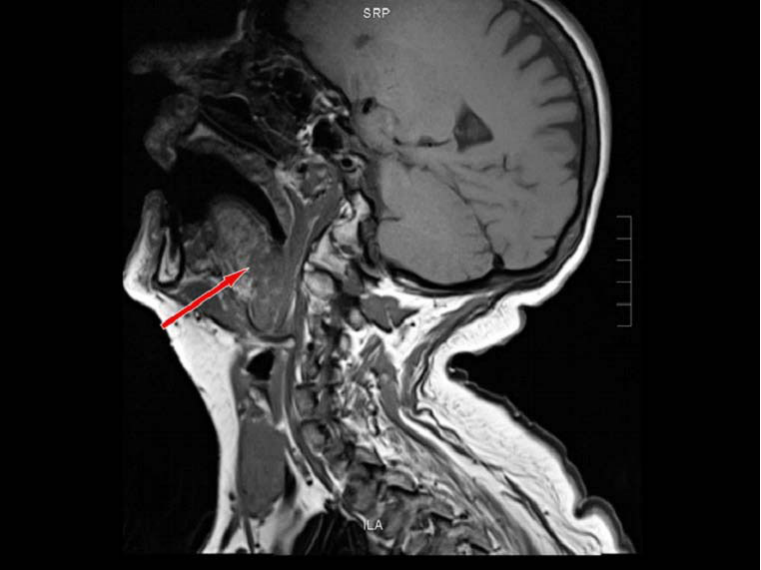
**Axial T2 Fat saturated MRI representing mass invading right base of tongue extending across the midline involving the right faucial tonsil**. Mass shows immediate T2 signal enhancement with bright focus within.

**Figure 4:**
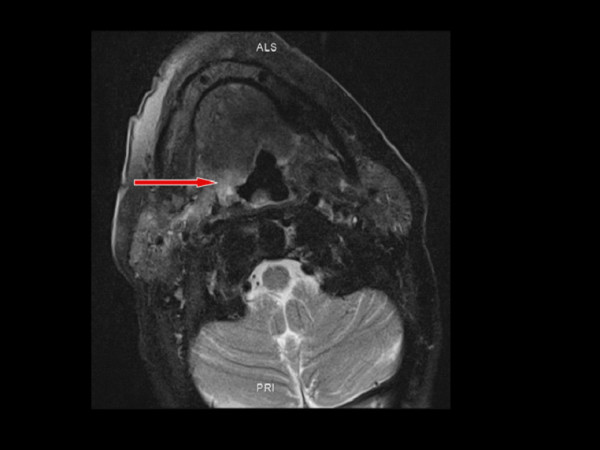
**Sagittal T1MRI, sagittal view, mass located at the base of the tongue**.

**Figure 5:**
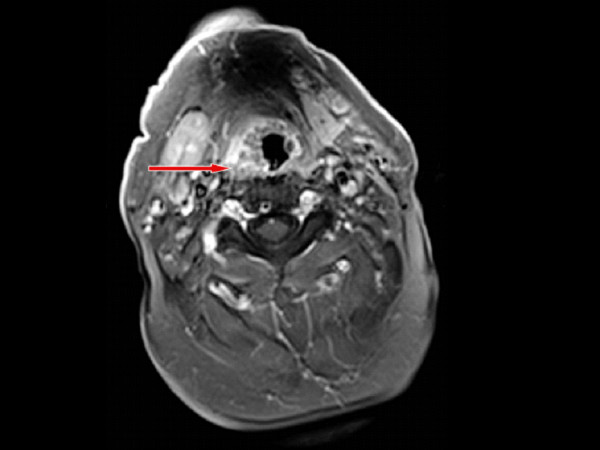
**Axial intermediate T1 Fat saturated MRI, post gadolinium on lesion on right base of tongue**. Lesion has irregular enhancement following gadolinium

An MRI taken shows an extensive BOT tumour across the midline which extends to involve the right faucial tonsil. The tumour has an intermediate T2 signal with foci of bright T2 fluid within (Figure 3). The intermediate T1 signal tumour shows irregular enhancement following Gadolinium (Figure 4, 5). The tumour appears exophytic. The tumour extended inferiorly to the level of the epiglottis which it displaced posteriorly. The vertical length of the tumour was 3.7 cm. Small (approx 1 cm) lymphadenopathy was noted bilaterally in level 2 but this was thought to be clinically unlikely to be involved with tumour in view of the size, morphological appearance and clinical nature of the disease. The appearances of the BOT tumour are not typical for the more common SCC in this location with the irregular bright T2 signal. However an unusual SCC appearance is still be more likely than a minor salivary gland rare tumour based a review of the literature.

**Figure 6:**
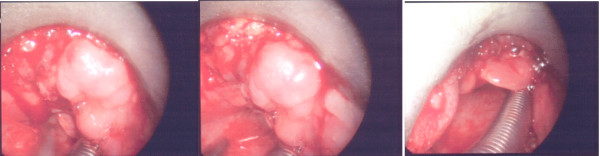
**Intraoperative views of the mass at the base of the tongue**.

**Figure 7:**
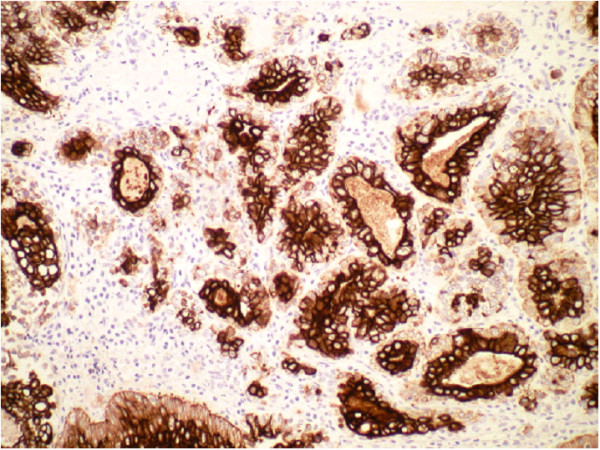
**AE1/AE3 Immunohistochemistry stain of epithelial-myoepithelial carcinoma**.

**Figure 8:**
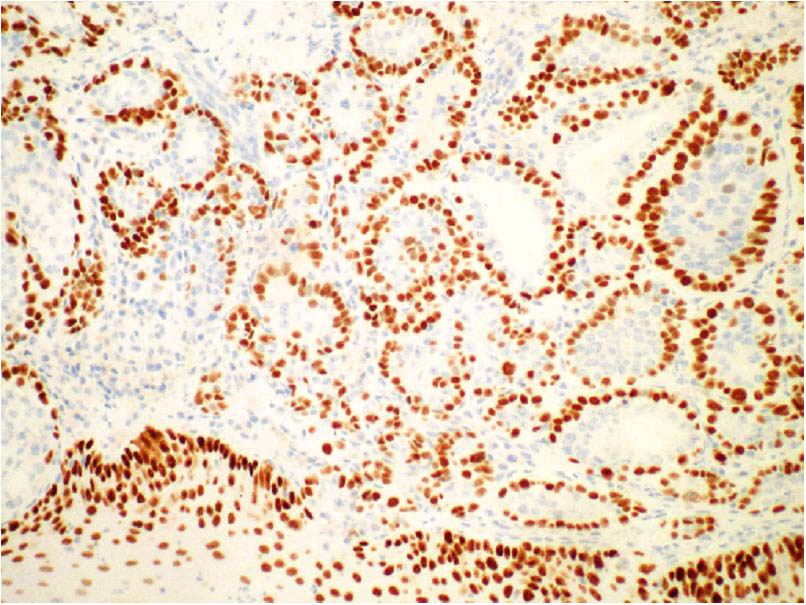
**p63 Immunohistochemistry stain of epithelial-myoepithelial carcinoma in the base of the tongue**.

**Figure 9:**
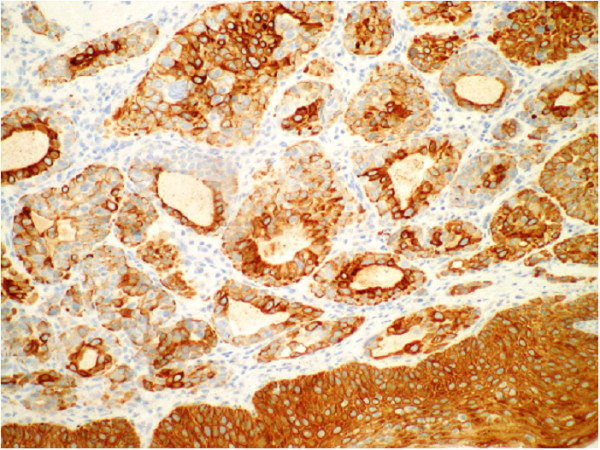
**CK5/6 Immunohistochemistry stain of epithelial-myoepithelial carcinoma, biopsy taken from mass at base of tongue**.

**Figure 10:**
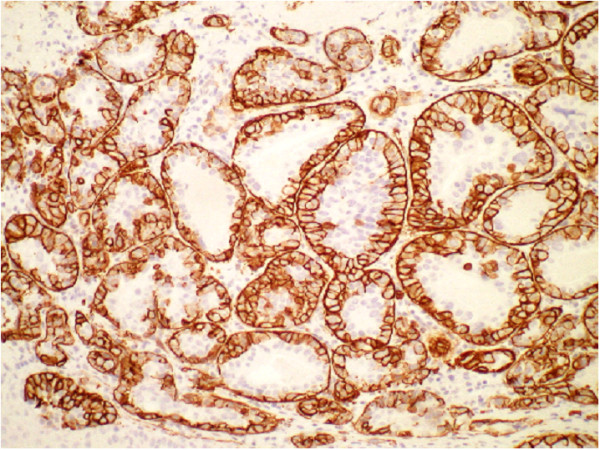
**SMA stain, Immunohistochemistry stain from mass at the base of tongue in a 60 year old female patient**.

A panendoscopy was performed which revealed a 4 cm submucosal lesion with a firm, posterior tongue (Figure 6). The mass extended over the midline, into the inferior half of the tonsil and into the vallecula but the glossal epiglottis was clear of tumour. The mass was not fixed to the mandible. Histological examination of the biopsy samples demonstrated epithelial-myoepithelial carcinoma of the tongue base. The morphology and the pattern of immunoreacitivity are typical for Epithelial-Myoepithelial carcinoma (Table 1 and Figures 7, 8, 9 and 10). The results of the panendoscopy and the biopsy were discussed with the patient and a thorough literature review enabled the multidisciplinary Head and Neck team to realistically discuss potential treatment options.

**Table 1:** Histology results for biopsy

IMMUNOHISTOCHEMISTRY	RESULT
p63	Positive in basal cells (nuclear)

CK5/6	Positive in luminal cell (cytoplasmic); positive in basal cell (Golgi)

HMWCK (CK34)	Positive in luminal cell (cytoplasmic); positive in basal cell (Golgi)

SMA	Positive in basal cells

AE1/AE3	Positive in luminal cells

Planning by the Radiation Oncology team was undertaken with a view to a 60 Gy treatment over 30 fractions with a 2 cm margin around the primary tumour. Her treatment was well tolerated although she did require daily hyoscine injections to dry up excessive secretions. She has had a complete clinical response and will be followed with a new MRI in 6 months.

## Literature Review

2 cases have been reported in the literature of epithelial-myoepithelial carcinoma in the base of the tongue [1,6] with contrasting modes of treatment (Table 2).

**Table 2:** Comparison of previous cases

	**Puri et al 2004 **[6]	**Kumai et al 2006 **[10]	Peters et al
**Patient**	Male 48 yo	Male 76 yo	Female 60 yo

**Base of Tongue Location**	Right	Left	Right

**Tumour Size**	50 mm × 30 mm	40 mm × 20 mm	37-15 mm

**Treatment Modality**	3 drug Chemotherapy (cisplatin, doxorubicin and 5-Fluorourcil) Radiotherapy (66 Gy) (Coblalt Teletherapy Unit)	Surgery (subtotal glossectomy, bilat neck dissection and rectus abdominis flap)	Radiotherapy, 60 Gy over 30 fractions, 2 cm margin

**Follow up**	Nil Recurrence (14 months)	Nil recurrence (19 months)	Complete clinical response

These two cases were both published as "world firsts" and whilst sharing a common histology of tumours in an identical location (base of tongue), were where managed in different ways. This allowed our team to present the patient with options for management of her rare tumour.

## Discussion

There are three major salivary glands--parotid, submandibular, and sublingual--as well as innumerable minor salivary glands distributed throughout the mucosa of the oral cavity and nasopharynx. All these glands are subject to inflammation or to the development of neoplasms [7]. These glands give rise of over 30 histologically distinct tumours [7].

The tumours in Table 3 represent only around 2% of human neoplasms and occur predominantly in the parotid gland (60-80%), followed by the submandibular gland (10%) and then the minor salivary glands (including the sublingual gland) [7]. Interestingly, the parotids have the lowest level of malignancy (15-30%), with the submandibular gland (40%) and the minor salivary gland having a 50% malignancy rate, whilst the rate of malignancy in the sublingual gland is 70-90% [7].

**Table 3:** Histologic Classification and Incidence of Benign and Malignant Tumours of the Salivary Glands [7]

BENIGN	MALIGNANT
Pleomorphic adenoma (50%) (mixed tumour)	Mucoepidermoid carcinoma (15%)

Warthin tumour (5% to 10%)	Adenocarcinoma (NOS) (10%)

Oncocytoma (1%)	Acinic cell carcinoma (5%)

Other adenomas (5% to 10%)	Adenoid cystic carcinoma (5%)

Basal cell adenoma	Malignant mixed tumour (3% to 5%)

Canalicular adenoma	Squamous cell carcinoma (1%)

Ductal papillomas	Other carcinomas (2%)

Epithelial-myoepithelial carcinomas are defined, histologically, by tubular or ductal structures lined by both basal myoepithelial cells and luminal epithelial cells similar to normal intercalated salivary gland ducts. Whilst neoplasms are monoclonal and, as such, comprise one cell type, there are uncommon tumours at all sites which show divergent differentiation. In some case, as for this tumour, this can be explained by the common precursor cells for both of the cell types represented in tis particular tumour.

Immunohistochemistry for expression of different cell proteins is essential in identifying the two cell types demonstrated in this tumour. The outer basal or myoepithelial cell layer expresses p63 (figure 8), low molecular weight cytokeratins (CK5/6) and variably expresses other cytokeratin subclasses because these cells unique in expressing both epithelial and smooth muscle characteristics reflecting their normal function of lining ducts AND being capable of contraction to aid salivary fluid flow. The inner luminal layer is typical of all epithelial cells in expressing cytokeratin but not smooth muscle proteins.

This tumour displayed the typical and characteristic features of Epithelial-Myoepithelial Carcinoma with an inner luminal epithelial layer (cytokeratin positive only Figures 7 &9) and an outer myoepithelial cell layer (cytokeratin positive Figure 9) and co-expression of smooth muscle proteins (Figures 8 &10).

Epithelial-myoepithelial carcinomas, whilst rare, most commonly occur in the parotid gland, but as with all salivary gland tumours, may arise anywhere in the naso-oro-pharynx in relation to minor salivary glands. By definition, an epithelial-myoepithelial carcinoma is a hybrid tumour, which is a tumour consisting of two distinctly different entities, each of which conforms to an exactly defined tumour category [5].

Epithelial-myoepithelial are, low grade malignant tumours although some tumours do exhibit more rapid growth and high grade behaviour. Histologically the high grade tumours show evidence of dedifferentiation which is not seen otherwise. Tumour behaviour can, therefore to some extent, be predicted by histologic features. In this case, the prediction would low grade malignancy.

During a study of 954 consecutive salivary gland tumours, Fonseca et al [8] found 22 epithelial-myoepithelial carcinomas (2.3%). In all these cases, the tumours were excised with the associated salivary gland. Follow up of these patients indicated a recurrence rate of 41% with a 5 year survival rate of 87.5% and a 10 year survival rate of 67.5%. This rate of recurrence was similar to what was observed by Corio et al (39%) [9].

A review of the literature has revealed only two previous instances at the base of tongue [6,10] which were treated with chemo-radiation and surgery respectively without recurrence over a year later. Surgery and chemo-radiation are the two treatment modalities reported in the literature to have been used in base of tongue tumours and it is extremely helpful in a tertiary referral centre to have instant access to the only published experience with this particular tumour in this location.

It is important for very rare cases such as this to be reported particularly in respect of behaviour and treatment so as to build a database for these tumours. The current trend in journals not to publish case reports not only discourages junior researchers from getting on the publishing ladder but, as in our patient's case, could have severely limits evidence based options. Journals not publishing case report include CA: *A Cancer Journal for Clinicians *(Impact factor 2007 69.26) and the Journal of the American Medical Association (JAMA - Impact factor 2007 25.5), both highly respected journals with high ranking ISI impact factors.

Whilst the authors do not advocate that every journal should practice publishing case reports that have been published multiple times in the past, we would argue that limiting the world literature to "world firsts" will be to the detriment of our patients.

## Competing interests

The authors declare that they have no competing interests.

## Authors' contributions

PP performed the literature review, participated in the clinical care of the patient and drafted the manuscript. CR drafted the "case for the case report" component of the manuscript and assisted with the overall manuscript and participated in the clinical care of the patient. JE performed the panendoscopy of the patient. PS assisted with the literature review and clinical care of the patient. LM reviewed the biopsy specimen histology and determined the diagnosis. BB directed the radiation therapy component of the treatment. SJ reviewed the MRI for technical radiology input. WC was the consultant surgeon in overall care of the patient. All authors read and approved the final manuscript.

## Consent

Written informed consent was obtained from the patient for publication of this case report and accompanying images. A copy of the written consent is available for review by the Editor-in-Chief of this journal.
